# Observation of Anatomical Structures in the Human Larynx Using Micro-Computed Tomography with Lugol’s Solution Enhancement

**DOI:** 10.3390/diagnostics13183005

**Published:** 2023-09-20

**Authors:** Kyu-Ho Yi, Siyun Lee, Ji-Hyun Lee, Hyung-Jin Lee

**Affiliations:** 1 Division in Anatomy and Developmental Biology, Department of Oral Biology, Human Identification Research Institute, BK21 FOURProject, Yonsei University College of Dentistry, 50-1 Yonsei-ro, Seoul 03722, Republic of Korea; kyuho90@daum.net; 2 Maylin Clinic (Apgujeong), Seoul 07335, Republic of Korea; 3 Department of Cancer Biology, Dana-Farber Cancer Institute and Harvard Medical School, Boston, MA 02215, USA; siyun@broadinstitute.org; 4 Department of Anatomy and Acupoint, College of Korean Medicine, Gachon University, Seongnam 13120, Republic of Korea; 5 Department of Anatomy, Catholic Institute for Applied Anatomy, College of Medicine, The Catholic University of Korea, Seoul 06591, Republic of Korea

**Keywords:** Lugol’s solution, micro-CT, three-dimensional reconstruction, larynx, whole-mount staining, anatomy

## Abstract

Histological and naked-eye dissections are frequently used to investigate human anatomy. However, limitations of conventional methods include tissue damage and difficulty in observing structures, rendering findings limited. Micro-computed tomography (micro-CT) allows for a three-dimensional observation with whole-mount staining for contrast enhancement. A precise anatomical understanding of the larynx is essential for both the medical and surgical fields; however, the larynx is difficult to dissect because of its minuscule and complex structures. Therefore, we aimed to clarify the detailed anatomy of the larynx using micro-CT. The study was conducted on twelve specimens of cadavers using Lugol-based-contrast micro-CT. Using Lugol-micro-CT, relevant information on human structures was obtained. Consequently, we successfully employed the Lugol-micro-CT technique in the analysis of specific human soft tissue structures that are challenging to analyze using conventional methods.

## 1. Introduction

The larynx is a delicate and complex structure of the human body, comprising an intricate cartilage skeleton bound by fibro-elastic membranes [1,2]. The minuscule intrinsic muscles of the larynx enable these cartilages to function primarily as a protective sphincter for the airway, playing a key role in vocalization [1,2]. Understanding the structure and function of the larynx is essential for practitioners and surgeons, as any pathology affecting this structure can lead to significant consequences [1,2]. For example, in neck trauma, where the area’s complexity and closeness to vital organs become critical, an accurate understanding of this anatomy is indispensable for clinicians [3].

Ultrasonography and micro-magnetic resonance imaging have become increasingly prevalent in ENT, particularly for studying and diagnosing the larynx [4,5,6,7,8]. Their benefits, from cost-effectiveness to safety, make them especially valuable in emergencies and during health crises such as the COVID-19 pandemic. Despite these advantages, there remains a significant gap in research focusing on accurately analyzing or depicting the larynx’s complex three-dimensional structure [4,5,6,7,8].

Anatomical research methods for observing structures without magnification, such as dissection, tissue staining, and imaging techniques like computed tomography (CT) and ultrasonography, come with inherent limitations [9,10]. While ultrasonography has demonstrated great promise, there remains a marked gap in research that accurately portrays the larynx’s intricate three-dimensional structure. Dissection risks damaging delicate structures, potentially resulting in inaccurate anatomical representations [11]. Moreover, once a dissection is performed, it is impossible to view the original morphology before tissue removal. Tissue staining, on the other hand, delivers just a two-dimensional cross-sectional image of a small tissue segment, offering limited insight into its relationship with adjacent structures [9,12,13,14,15]. And while high-resolution imaging equipment is undeniably effective, it often struggles to capture the larynx’s finer details [11].

To surmount these challenges, we explored the advanced imaging technique of micro-computed tomography (micro-CT) [9,11,13,14,15]. Micro-CT, which leverages X-rays to produce intricate three-dimensional images of small samples, presents a potential resolution. Owing to its high resolution and prowess in capturing fine details, micro-CT is ideally suited for examining the intricate internal structures of biological specimens [9,11,13,14,15], including the larynx. Micro-CT offers enhanced visualization of soft tissue structures with contrast agents like phosphotungstic acid and Lugol’s iodine.

Micro-CT works similarly to traditional CT scanning used in medical imaging. It involves taking a series of X-ray images of the sample from different angles and then using computer algorithms to reconstruct them into a 3D representation. The resulting images allow researchers to visualize and analyze the sample’s internal structures, density variations, and spatial relationships.

Micro-CT has applications in various fields, including biology, materials science, paleontology, archaeology, and engineering. It provides non-destructive imaging that allows researchers to study the internal structures of objects without the need for physical dissection or sectioning. Its high resolution and ability to visualize hard and soft tissues make micro-CT a valuable tool for understanding the internal anatomy and properties of a wide range of objects and specimens.

Micro-CT has two main advantages. First, the structural damage is minimal, and microscopic anatomical structures can be observed and analyzed in three dimensions. Whole-mount staining has been used in many previous studies to reveal minuscule structures [16] safely. Second, various contrast agents (phosphotungstic acid and Lugol’s iodine) can be used to visualize soft tissues that are difficult to observe with general CT [16]. This contrast helps micro-CT with deeper penetration imaging of small objects and specimens due to micro-CT having limited penetration depth. Larger or denser samples may not allow X-rays to penetrate effectively, resulting in a reduced image quality or incomplete visualization of internal structures. Also, using contrast can help achieve higher resolution in micro-CT images, which often comes at the expense of a smaller field of view. This means that while fine details can be captured, the overall size of the sample that can be imaged in high resolution may be limited. Micro-CT with contrast media images can sometimes be affected by artifacts, which are discrepancies between the reconstructed image and the true sample structure. These artifacts can arise from factors such as sample movement during imaging, beam hardening due to varying tissue densities, and limitations in the imaging hardware. While micro-CT is excellent for visualizing hard tissues like bones, teeth, and materials, it may have limitations in distinguishing soft tissues with similar densities. Contrast agents or staining techniques might be needed to enhance the visibility of soft tissue structures.

Phosphotungstic acid and Lugol’s iodine can be used as contrast agents along with micro-CT; the choice between phosphotungstic acid and Lugol’s iodine depends on the type of sample and the specific structures researchers aim to visualize. Phosphotungstic acid is preferred for imaging mineralized structures, while Lugol’s iodine is more suitable for enhancing the contrast of soft tissues. Researchers often choose the appropriate contrast agent based on their imaging goals and the nature of the sample being studied.

In previous studies, small animals, embryonic vertebrates, and minuscule parts (tissue size of 1 to 4 cm) of the human body were studied using micro-CT imaging [9,12,13,14,15]. The larynx is a delicate and complex human body structure comprising an intricate cartilage skeleton held together by fibro-elastic membranes [1,2]. Miniscule intrinsic muscles of the larynx moving these cartilages allow them to play a vital role as a protective sphincter of the airway, including vocalization. Insight into the structure and function of the larynx is critical for practitioners and surgeons, as pathology in this structure significantly impacts consequences.

Capitalizing on these attributes, our study endeavors to shed light on the anatomical intricacies of the larynx—structures traditionally challenging to study without magnification—employing micro-CT in tandem with whole-mount Lugol’s staining.

## 2. Materials and Methods

This study was conducted in compliance with the Act on Dissection and Preservation of Corpses of the Republic of Korea (act number: 14885) and approved by the Institutional Cadaver Research Committee of the College of Medicine, the Catholic University of Korea (MC22SISI0098). All donors or authorized representatives provided written informed consent for the use of the cadavers and consent for their use in future research on the related materials.

Ten embalmed cadavers (seven males and three females; mean age, 72.1 years) were dissected to reveal the laryngeal muscles and surrounding neurovascular structures and utilized for micro-CT imaging [16]. We obtained the appropriate consent to use cadavers that had been legally donated to the Department of Anatomy, Catholic University of Korea. The cadavers had no trauma, surgery, or deformity of the neck region. Dissection was conducted by carefully removing the skin, subcutaneous tissue, deep fascia, muscles, and superficial vessels. The laryngeal structures were exposed to examine the possible anatomical structures. Then, Lugol’s staining was performed.

### 2.1. Lugol’s Staining

After the dissection was conducted and specimens were obtained, they were fixed for 3 days by immersing in 10% normal buffered formalin [13]. Then, the specimens were dehydrated for another 3 days with increasing concentrations of EtOH from 30% to 70%. Thereafter, the specimens were stained in 3% Lugol’s solution in 70% ethanol for 10 days. Lugol’s solution contains one part iodine and two parts potassium iodide in an aqueous solution. To obtain a concentration of 3%, 1% iodine and 2% potassium iodide were dissolved in double-distilled water. All instances of Lugol staining performed in the course of this study were performed in 70% EtOH and mixed according to the above ratio.

### 2.2. Micro-Computed Tomography

The data were analyzed using a Nano & Microfocus X-ray CT (v|tome|x m 240, General Electrics) with minimum detectability of 0.2 μm, voxel resolution of 240 kV, and image resolution of image pixel grid of 2240 × 2240. The three-dimensional (3D) reconstructed model was analyzed with a 3D slicer (v.5.1.0, Slicer Community, www.slicer.org accessed on 27 January 2023), which is an open-source development platform used in medical and biomedical applications.

## 3. Result

In this study, to analyze the structures using micro-CT, we consulted with an otolaryngology specialist, and the findings were cross-validated by three anatomists. Despite the significant drawback of micro-CT being unable to represent natural color, it enabled the observation of most anatomical structures in their preserved state that would otherwise be damaged during gross dissection. The 3D models obtained through micro-CT provided a clear visualization of most muscular structures, blood vessels, and nerves, facilitating a more comprehensive understanding of their three-dimensional spatial relationships with the surrounding structures. Furthermore, several advantages of the 3D models obtained through micro-CT were discussed. All anatomists agreed on the benefits of micro-CT, highlighting its ability to circumvent labor-intensive and time-consuming dissections and emphasizing the advantage of having digitized models that can be revisited and reviewed at any time.

**Figure 1:**
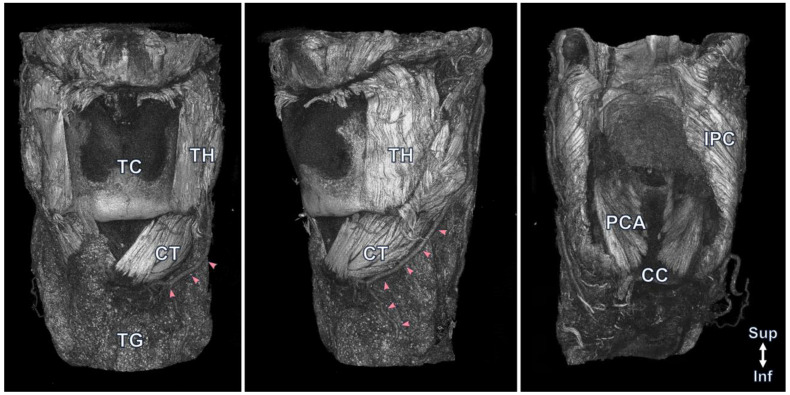
The 3D model by micro-CT provides anterior, lateral, and posterior views of the external anatomical structures of the larynx. Externally, the hyoid bone, thyroid cartilage (TC), thyroid gland (TG), cricothyroid muscle (CT), thyrohyoid muscle (TH), posterior cricoarytenoid muscle (PCA), inferior pharyngeal constrictor muscle (IPC), and superior laryngeal artery, which is indicated with pink arrowheads, and even muscle fibers are well observed.

**Figure 2:**
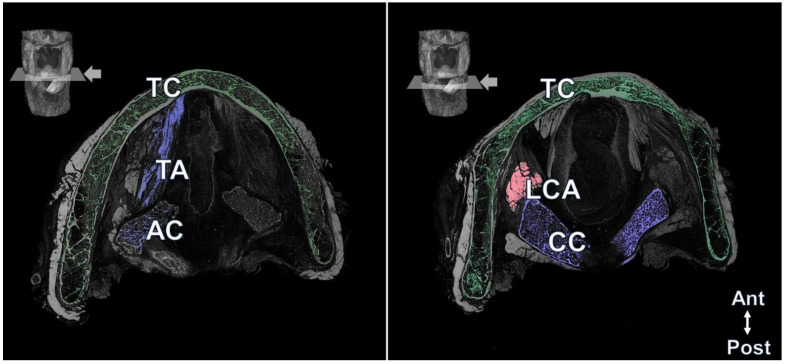
The transverse view of the internal laryngeal structures. Lower left and right transverse images were obtained from the level indicated with pink lines and arrows in the upper larynx photograph. The thyroarytenoid muscle fibers were well observed between the thyroid and arytenoid cartilages in the left image. The lateral cricoarytenoid muscle (LCA), which is indicated in pink, was identified at the lateral side of the cricoid cartilage (CC, Blue), which is indicated in violet, and the posterior cricoarytenoid muscle (PCA) posterior to the CC. Ant, anterior; Post, posterior; TC, thyroid cartilage (Green).

Most of the minuscule larynx structures were observed in the 3D reconstruction. Externally, the hyoid bone, thyroid cartilage, cricothyroid muscle, thyrohyoid muscle, surrounding neurovascular structures, and even muscle fibers were observed (Figure 1). In the transverse view, the thyrohyoid muscle was identified between the hyoid bone and thyroid cartilage, and the transverse and oblique arytenoid muscles located between the arytenoid cartilage and both arytenoid cartilages were observed. The thyroarytenoid muscle was observed between the posterior surface of the thyroid cartilage and arytenoid cartilage at the transverse level of the cricoid cartilage. The lateral cricoarytenoid muscle running anteriorly just below the arytenoid cartilage was also observed (Figure 2). Blood vessels running in various directions were also observed in the larynx. Detailed information on the 3D reconstruction model of Lugol’s solution-based method can be found in Supplemental Videos S1 and S2.

## 4. Discussion

The present study aimed to evaluate the availability of Lugol’s solution in the entire human larynx. When contrasted with observations made with the naked eye, the 3D model of the larynx rendered by micro-CT provided a significantly more detailed and precise depiction, particularly in areas such as the vocal folds, intricate individual muscle morphologies, and the relationships between these muscles.

Most recent morphological studies performed PTA staining on small human samples such as the infraorbital area (e.g., anatomy of the orbicularis retaining ligament) and the area of the nasolabial folds (e.g., muscular arrangement). In a PTA-applied micro-CT study, the minuscule muscular structures of the nasolabial fold area and the original location of the orbicularis retaining ligament and its course within the muscular and connective tissue were well distinguished because the size of the specimens was small. Although the sample size was relatively larger than the specimens used in previous studies, the external and internal muscular structures of the larynx were also well observed in the present study. Although PTA has the advantage of positive interaction with collagen in the analysis of muscles, ligaments, and tendinous structures, Lugol’s solution can also be effectively applied to human samples to observe muscle and neurovascular structures [10].

The larynx is composed of a complex skeleton of cartilage and fibro-elastic membranes. Laryngeal intrinsic muscles moving these cartilages play a fundamental role in protecting the sphincter of the airway, including vocalization. Understanding the structure of the larynx is important for both the surgical and medical fields, as pathology can significantly impact patients [1,2].

Naked-eye dissections are often used to study human anatomy, but their drawback includes damage to the soft tissue, making it difficult to observe its structures. Our research using Lugol-micro-CT identified relevant information on the human larynx.

This result would provide key information in the treatment of laryngeal diseases, convulsive dysphonia, adductor spasmodic dysphonia, and laryngeal dystonia, causing vocal tremors [17]. They are characterized by a broken or trembling voice during vocalization. When botulinum toxin is injected into the thyroarytenoid and lateral cricoarytenoid muscles that modulate the vocal cords, it is possible to provide 3D anatomical information regarding these muscles.

In addition, vocal cord injection, which is a micro-procedure for unilateral vocal fold paralysis, glottic insufficiency in functional dysphonia, presbyphonia, or vocal fold scarring, is a delicate procedure conducted based on anatomy [18,19]. Vocal cord immobility and paralysis, scars in the vocal cords, and age-related voice changes are indications for filler injection. When both sides of the vocal cords do not come in contact with each other, the voice becomes dysfunctional; hence, a temporary procedure to narrow the distance between the vocal cords using fillers is often conducted [20]. When approaching the vocal cords, it is possible to provide information on the path and location where the needle can safely access the vocal cords by analyzing the location of the thyroid cartilage, cricoid cartilage, and vocal cords in three dimensions [21]. The present study used cadaveric specimens, which is considered a limitation, unlike general CT or other imaging devices.

The current study has its limitations. Despite consistent anatomical structures of the larynx being observed throughout our research, a small sample size was utilized, leading to potential biases, particularly in accounting for morphological variations. In this study, we exclusively employed Lugol’s solution as the contrast-enhancing agent to showcase the 3D anatomy of the larynx via micro-CT. However, other contrast agents, such as phosphotungstic acid, might offer alternative benefits. Standardizing the staining process using Lugol’s solution across diverse specimens would enhance its applicability in various research settings. However, despite the improvements made in this research, the equipment and staining processes employed remain both time-intensive and costly. Future research should compare different staining agents to ascertain the most suitable one for optimal visualization.

## 5. Conclusions

In conclusion, 3D micro-CT images and observations meticulously proved the actual topography of the intricate larynx. We confirmed that the thyroid, cricoid, arytenoid cartilage, thyrohyoid, transverse and oblique arytenoid muscles, thyroarytenoid muscle, and lateral cricoarytenoid muscles with various blood vessels were observed in the larynx. Currently, clinical information regarding the minuscule anatomical structures of the larynx is lacking. Understanding the structure of these muscles could assist clinicians in further investigations and medical procedures. Through this study, it was confirmed that the entire larynx was visualized using micro-CT, and it was confirmed that Lugol’s solution is an appropriate contrast reagent in acquiring 3D information.

## Data Availability

Not applicable.

## Supplementary Materials

The following supporting information can be downloaded at: https://www.mdpi.com/article/10.3390/diagnostics13183005/s1, Video S1: Three-dimensional (3D) reconstruction model of the larynx. In the 3D larynx model, the transverse plane was moved serially from the superior to the inferior direction to observe the external and internal laryngeal structures. Arterial structures enter the thyrohyoid membrane and divide into several branches to be distributed to the internal laryngeal structures. In addition, easily distinguishable muscle fibers can be observed, including the thyroarytenoid muscle, cricothyroid muscle, lateral cricoarytenoid muscle, and posterior cricoarytenoid muscles; Video S2: Three-dimensional reconstruction model of the larynx with a rotational view of the other surface. The external structure of the larynx was also observed. The hyoid bone, thyroid cartilage, thyroid gland, cricothyroid muscle, thyrohyoid muscle, posterior cricoarytenoid muscle, inferior pharyngeal constrictor muscle, and superior laryngeal artery can be well observed.

## References

[1] Constable J.D., Bathala S., Ahmed J.J., McGlashan J.A. (2017). Non-recurrent laryngeal nerve with a coexisting contralateral nerve demonstrating extralaryngeal branching. Case Rep..

[2] Sperandio F.A., Imamura R., Tsuji D.H., Sennes L.U. (2016). Surgical approach to the thyroarytenoid branch of the inferior laryngeal nerve through the thyroid cartilage. Acta Cirúrgica Bras..

[3] Severin F., Rosu A.-M., Tiglis M., Checherita L.-E., Stegaru G., Cobzeanu M.D., Hainarosie R., Cobzeanu B.M., Palade O.D. (2023). Multidisciplinary Therapeutic Management in Complex Cervical Trauma. Medicina.

[4] Cergan R., Dumitru M., Vrinceanu D., Neagos A., Jeican I.I., Ciuluvica R.C. (2021). Ultrasonography of the larynx: Novel use during the SARS-CoV-2 pandemic. Exp. Ther. Med..

[5] Costache A., Dumitru M., Anghel I., Cergan R., Anghel A.G., Sarafoleanu C. (2015). Ultrasonographic anatomy of head and neck-a pictorial for the ENT specialist. Med. Ultrason..

[6] Gambardella C., Offi C., Romano R.M., De Palma M., Ruggiero R., Candela G., Puziello A., Docimo L., Grasso M., Docimo G. (2020). Transcutaneous laryngeal ultrasonography: A reliable, non-invasive and inexpensive preoperative method in the evaluation of vocal cords motility—A prospective multicentric analysis on a large series and a literature review. Updates Surg..

[7] Chen T., Chodara A.M., Sprecher A.J., Fang F., Song W., Tao C., Jiang J.J. (2012). A new method of reconstructing the human laryngeal architecture using micro-MRI. J. Voice.

[8] Elders B.B., Hermelijn S.M., Tiddens H.A., Pullens B., Wielopolski P.A., Ciet P. (2019). Magnetic resonance imaging of the larynx in the pediatric population: A systematic review. Pediatr. Pulmonol..

[9] Cho T.-H., Kwon H.-J., Jehoon O., Cho J., Kim S.H., Yang H.-M. (2022). The pathway of injectate spread during thoracic intertransverse process (ITP) block: Micro-computed tomography findings and anatomical evaluations. J. Clin. Anesth..

[10] De Bournonville S., Vangrunderbeeck S., Kerckhofs G. (2019). Contrast-enhanced microCT for virtual 3D anatomical pathology of biological tissues: A literature review. Contrast Media Mol. Imaging.

[11] Swart P., Wicklein M., Sykes D., Ahmed F., Krapp H.G. (2016). A quantitative comparison of micro-CT preparations in Dipteran flies. Sci. Rep..

[12] Dunmore-Buyze P.J., Tate E., Xiang F.l., Detombe S.A., Nong Z., Pickering J.G., Drangova M. (2014). Three-dimensional imaging of the mouse heart and vasculature using micro-CT and whole-body perfusion of iodine or phosphotungstic acid. Contrast Media Mol. Imaging.

[13] Gignac P.M., Kley N.J. (2014). Iodine-enhanced micro-CT imaging: Methodological refinements for the study of the soft-tissue anatomy of post-embryonic vertebrates. J. Exp. Zool. Part B Mol. Dev. Evol..

[14] Hur M.-S., Lee S., Kang T.M., Oh C.-S. (2021). The three muscle layers in the pyloric sphincter and their possible function during antropyloroduodenal motility. Sci. Rep..

[15] Hur M.S., O J., Yang H.M., Kwon H.J., Lee S., Lim H.S., Lim S.Y., Oh C.S. (2020). Heights and spatial relationships of the facial muscles acting on the nasolabial fold by dissection and three-dimensional microcomputed tomography. PLoS ONE.

[16] Jehoon O., Kwon H.J., Cho T.H., Woo S.H., Rhee Y.H., Yang H.M. (2021). Micro-computed tomography with contrast enhancement: An excellent technique for soft tissue examination in humans. PLoS ONE.

[17] Cannito M.P., Kahane J.C., Chorna L. (2008). Vocal aging and adductor spasmodic dysphonia: Response to botulinum toxin injection. Clin. Interv. Aging.

[18] Remacle M., Lawson G. (2007). Results with collagen injection into the vocal folds for medialization. Curr. Opin. Otolaryngol. Head Neck Surg..

[19] Anghel A.G., Anghel I., Dumitru M., Soreanu C.C. (2012). Respiratory and phonatory impairment due to iatrogenic vocal fold paralysis and paresis. A retrospective study of 188 patients. Rom. J. Leg. Med..

[20] Lee Y.C., Pei Y.C., Lu Y.A., Chung H.F., Li H.Y., Lee L.A., Fang T.J. (2021). Long-Lasting Effect after Single Hyaluronate Injection for Unilateral Vocal Fold Paralysis: Does Concentration Matter?. Biomolecules.

[21] DeFatta R.A., Chowdhury F.R., Sataloff R.T. (2012). Complications of injection laryngoplasty using calcium hydroxylapatite. J. Voice.

